# Validation of the Vivago Wrist-Worn accelerometer in the assessment of physical activity

**DOI:** 10.1186/1471-2458-12-690

**Published:** 2012-08-22

**Authors:** Jérémy Vanhelst, Rémy Hurdiel, Jacques Mikulovic, Gilles Bui-Xuân, Paul Fardy, Denis Theunynck, Laurent Béghin

**Affiliations:** 1 Centre d’Investigation Clinique, CIC-PT-9301-Inserm-CH&U, 59037, Lille, France; 2 Unité Inserm U995, Université Lille Nord de France, Lille, France; 3 ER3S, EA4110, Université Lille Nord de France, Dunkerque, France; 4 ER3S, Université d'Artois, Villeneuve d'Ascq, France; 5 Department of Family, Nutrition, and Exercise Sciences (FNES) Queens College, Flushing, New York, USA

**Keywords:** Cut-off, Accelerometry, Exercise, Validation, Calibration

## Abstract

**Background:**

Most accelerometers are worn around the waist (hip or lower back) to assess habitual physical activity. Wrist-worn accelerometers may be an alternative to the waist-worn monitors and may improve compliance in studies with prolonged wear. The aim of this study was to validate the Vivago® Wrist-Worn Accelerometer at various intensities of physical activity (PA) in adults.

**Methods:**

Twenty-one healthy adults aged 20–34 years were recruited for the study. Accelerometer data and oxygen uptake (VO_2_) were measured at sedentary, light, moderate and vigorous levels of PA.

**Results:**

Activity categories and accelerometer counts were: sedentary, 0–15 counts·min^−1^; light, 16–40 counts·min^−1^; moderate, 41–85 counts·min^−1^; and vigorous activity, >; 85 counts·min^−1^. ANOVA repeated measures was used to determine the relationship between accelerometry data output and oxygen consumption (r = .89; p <; .001). The Bland and Altman method showed good agreement in the assessment of energy expenditure between the indirect calorimetry and the data obtained by the accelerometer.

**Conclusions:**

Results of the study suggest that the Vivago® wrist-worn accelerometer is a valid measure of PA at varying levels of intensity. The study has also defined threshold values at 4 intensities and hence te Vivago® accelerometer may be used to quantify PA in free living conditions among adults. This device has possible application in treating a variety of important health concerns.

## Background

Obesity in children and adults, coupled with adverse health consequences, has increased dramatically in recent years resulting in an urgent need for population-based interventions. The ability to prescribe exercise accurately is an important consideration in developing intervention programs [1]. Many methods are available to assess physical activity (PA) in free living conditions (FLC), e.g. PA questionnaires, self reports, indirect calorimerty, pedometry, and accelerometry [2-4]. PA questionnaires are often used because they are low cost, easily administered, and can assess large numbers simultaneously. However, questionnaires are subjective and have problems of reliability and validity [4-6]. Generally, the questionnaire tends to overestimate PA and can lead to misclassification of subjects [6]. Pedometers provide an inexpensive and reliable objective measure of activity by counting the number of steps performed by the subject per day. However, the pedometers are not able to quantify PA levels. Accelerometry represents an objective, inexpensive and non invasive method to measure PA [7]. The advantages of accelerometry are precision, accuracy, small size, light weight and ease of use. Accelerometry technology is based on mechanical principles incorporating a piezoelectric element and a seismic mass housed in a device called an accelerometer, which measure accelerations. Acceleration is defined as the change in velocity over time; it measures the quantity and intensity of movement. When the subject moves, the sensor undergoes acceleration, and the seismic mass causes the piezoelectric element to experience deformation in the form of bending, direct tension or compression. These conformational changes cause a displaced charge to build up on one side of the sensor, which can generate a variable output voltage signal that is proportional to the applied acceleration. This voltage signal, after being filtered and amplified, is then sampled at a prefixed frequency by the device to convert the analog voltage signal to a digital series of numbers, which are called “counts” [8]. Most accelerometers are usually worn around the waist, hip or lower back with an elastic belt and adjustable buckle. The Vivago® accelerometer ( http://www.vivagowellness.fr) is a wrist-worn accelerometer, which, when compared with waist-worn monitors may be more convenient to wear and may lead to improved compliance for studies where there is prolonged wear (usually 7 d for assessing PA habitual). To our knowledge, no published studies have assessed the validity of the Vivago® wrist-worn accelerometer for assessing the PA in FLC.

Therefore, the purpose of the present study is to measure the validity of the Vivago® wrist-worn accelerometer in the assessment of PA and to define thresholds for detecting different levels of PA.

## Methods

### Subjects

Twenty-one healthy and active adults (10 females and 11 males), aged 20–34 yr, were recruited for the study. The mean ± SD for age, body mass, and height were 29.3 ± 5.1 years, 79.6 ± 12.3 kg, and 180.2 ± 9.1 cm, respectively. The purpose and objectives of the study were explained to each subject before the study began and written informed consent was obtained. The study was approved by the Lille University Research Ethics Committee (Comité de Protection des Personnes, Lille, France). All procedures were performed in accordance with the ethical standards of the Helsinki Declaration of 1975, as revised in 2008, and Good Clinical Practice [9].

### Procedures

All subjects were required to undergo a physical examination to exclude pathologies that might have caused subjects to be excluded. Eligibility criteria included body mass index between 19 and 24.4 kg/m^2^, 18 to 35 years of age, and normal clinical examination including normal psychomotor development. Exclusion criteria included obesity, chronic diseases, cardiovascular or metabolic diseases. Body mass was measured without shoes and heavy outer garments to the nearest 0.1 kg using an electronic scale (Oregon Scientific®, GA 101, USA). Height was measured without shoes to the nearest 0.1 cm using a standard physician’s scale. PA was assessed by accelerometry and oxygen uptake by gas-collection methodology. Intensity of PA varied from sedentary to vigorous. Activities were selected that reflected typical PA in adults under normal living conditions, e.g. walking, running and sitting in the office or resting. Treadmill speed was similar to the study of Freedson et al. [10]. Subjects performed six consecutive 10 minute periods of activity at increasing levels of intensity from sedentary to vigorous. Intensities of activity were defined as sedentary (resting on a bed, and reading a book), light (walking slowly at 2.5 mph); moderate (walking at 3.7 mph and running slowly at 5 mph); and vigorous (running at 6.2 mph). A treadmill (Marquette 2000, SOMA Technology®, Cheshire, USA) was used to represent light, moderate and vigorous PA. All intensities were performed during the same testing session with a rest period between each activity varying between 3 and 10 minutes according to the fatigue status of participant. The criterion chosen to restart the next activity was to recover a respiratory quotient of rest (± 10% from rest period). All tests were performed at the University Applied Physiology Laboratory.

### Materials

#### Vivago**®** accelerometer

Vivago® is an accelerometer worn at the wrist of the subject (Vivago Wellness®, Paris, France). The activity signal, which is constructed from measured force changes at the unit’s movement sensor, is continuously recorded on average once per minute and can store data recorded at 1 minute epoch. The Vivago® wrist-worn accelerometer reacts to omnidirectional changes in acceleration which generate a voltage via a piezoelectric sensor. The signal is amplified, digitized, and stored in memory as activity counts. The dynamic range of the accelerometer is ± 4 G. The sample rate for body motion is 40 samples per second. The Vivago® accelerometer is sensitive to movements in the 0.5–10 Hz range. The same accelerometer was used for all subjects. Data were downloaded from the monitor to a computer after completion of all activities. Accelerometer data between minutes 3 and 10 were used to represent physiologic steady state. Data were expressed as the mean in *counts·min*^*−1*^.

#### Indirect Calorimetry

Oxygen consumption (VO_2_) and carbon dioxide production (VCO_2_) were measured every 10 s for 10 min during each activity using a gas analyzer (Respironics Novametrix Medical System® inc, NICO 7300, Wallingford, USA and Datex®, Ohmeda, USA). The gas analyzer was calibrated with standard gases before each session and was synchronized with the accelerometer. Subjects wore an adapted mask that was connected by plastic tubing to the gas analyzer. The mask was worn during all experiments. Data captured between minutes 3 and 10 of each activity level were analyzed.

### Statistical analysis

Data were analyzed using the Statistical Package for the Social Sciences, Windows 11.5 (SPSS Inc., Chicago, USA, IL) and Excel 2003 (Microsoft Inc., Redmond, USA, WA). Receiver operator curves (ROC) were used to determine the Vivago® value that best distinguished one level of intensity from another. The first was used to distinguish sedentary from light PA data. The threshold was defined as the maximum receiver operator curves value for sedentary PA. To define the lowest value of the subsequent PA level (i.e. light physical activity), we added one count to the preceding threshold. For example, if the threshold of the receiver operator curve for distinguishing sedentary from light PA was 15, the range for sedentary PA was defined as 0–15. The next threshold was 16 and corresponded to the lowest value for light PA. This procedure was repeated twice to define ranges for light, moderate, and vigorous intensities. Individual data were pooled to defined thresholds. Repeated-measures analysis of variance (ANOVA) was used to correlate oxygen consumption with accelerometer data for the whole study population. The Bland & Altman method was also used to compare the energy cost assessed by the indirect calorimetry and accelerometry [11]. Heteroscedasticity was tested by the « Levene » Test for equality of variances.

## Results

### Vivago**® accelerometer thresholds**

Three ROCs were plotted according to the intensity of PA. Thresholds were identified as sedentary, light, moderate and vigorous activity. Thresholds of 15 counts. min^−1^ between sedentary and light intensity, 40 counts. min^−1^ between light and moderate intensity, and 85 counts. min^−1^ between moderate and vigorous intensity were identified. Therefore, the values ranges for sedentary, light, moderate, and vigorous activities for the Vivago® accelerometer were 0–15, 16–40, 41–85, and 85 counts.min^−1^, respectively.

### Accelerometer counts, oxygen consumption and MET values

Table 1 includes the accelerometer counts, oxygen consumption and MET values derived from calorimetry indirect and accelerometry, expressed both in minutes for each intensity. The chosen physical activities provided a wide range in MET values (1.6–8.7) with a correspondingly wide range in accelerometer counts (6.5–95.6 counts·min^−1^).

**Table 1:** Counts and oxygen uptake at each physical activity intensity [mean (± SD)]

	**Sedentary**	**Light**	**Moderate**	**Vigorous**
Accelerometer (counts·min^−1^)	6.5 (5.5)	18.3 (2.8)	45.9 (8.7)	95.6 (5.3)
Oxygen uptake (ml.kg.min^−1^)	5.7 (1.1)	14.2 (1.7)	24.4 (5.9)	37.1 (6.3)
MET score^†^	1.6 (0.3)	3.9 (0.4)	5.6 (1.8)	9.1 (1.7)
MET score*	1.6 (0.3)	3.2 (0.5)	5.2 (1.7)	8.7 (1.6)

^†^ MET value derived from indirect calorimetry.

* MET score derived from data accelerometer.

**Figure 1:**
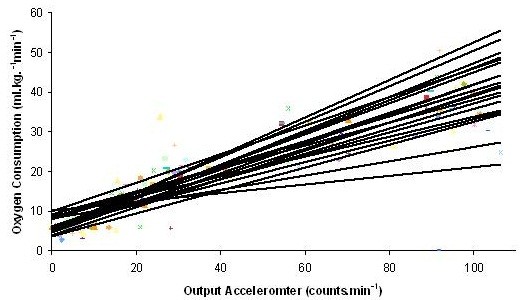
Individual regression plots for the change in oxygen consumption and output accelerometer during all activities (n = 21).

**Figure 2:**
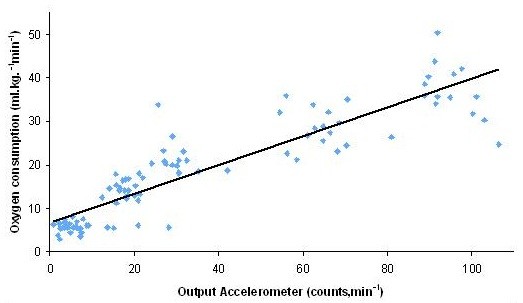
Correlation between output accelerometer and oxygen consumption (n = 21).

Oxygen uptake, data output from the accelerometer and MET values increased with increasing exercise intensity (Table 1; Figures 1 &2). Within-individual, between-individual, and overall correlations for vector magnitude and oxygen consumption are presented in Table 2. Individual r-values between PA level, output accelerometer and oxygen uptake exceeded 0.9 in all cases.

**Table 2:** Correlation coefficients for output accelerometer and oxygen consumption

	**Within**	**Between**	**Overall**
Correlation coefficient	0.94	0.92	0.89

Homoscedasticity was found for the variables because the variance was very closed. When pooling data from all participants, a significant correlation was found between output accelerometer and oxygen consumption (R = .89, P <; .001; ANOVA; Figure 2). Table 1 shows that the physical activity levels were associated with a wide range of oxygen uptakes (5.7–37.1 ml.kg^−1^.min^−1^). Oxygen consumption was significantly higher at increased PA intensities.

**Figure 3:**
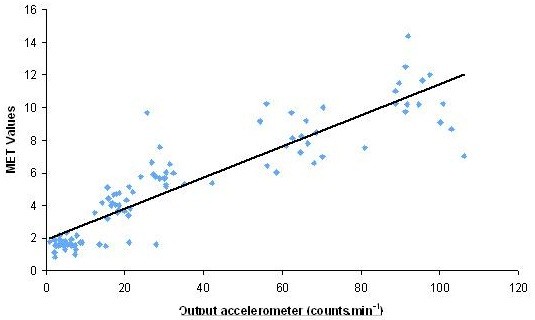
Regression line for MET score versus accelerometer (Vivago) counts.

Figure 3 shows the regression lines for MET score versus accelerometer counts. The formula estimating MET score using counts is:

(1) $$\text{MET}=0.0949\times \left(\text{counts}.\min^{-1}\right)+1.9145$$

The mixed model concordance correlation coefficient, corresponding to an R^2^ in standard linear regression, was 0.82.

**Figure 4:**
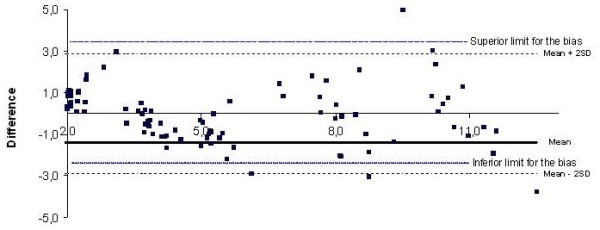
Difference MET values between indirect calorimetry and accelerometer output in 21 healthy volunteers.

Figure 4 shows a good agreement in the assessment of MET values between indirect calorimetry and output accelerometer because the mean difference was within the limits of agreement and most data points were within the limits of agreement of bias (Bland & Altman). The mean difference was very close of 0 (1.1 ± 1.3), and the limits of agreement were −2.9 to 2.9.

## Discussion

Accelerometry is a useful tool for prescribing exercise to help develop PA intervention programs. New accelerometers need to be tested for validity and reliability. Accelerometers most often used in epidemiological and clinical studies previously have been calibrated and validated for assessing PA patterns [12-17]. The Vivago® wrist-worn accelerometer has been validated and may be used in long-term monitoring of sleep/wake patterns with similar performance to actigraphy [18]. However, no published studies have been performed on the validity of the Vivago® wrist-worn accelerometer in the assessment of PA in FLC.

Even if many movements during activities of FLC may not be recorded with a wrist-worn accelerometer, Zhang et al. demonstrated [19] that the ability to detect certain types of PA with a wrist-worn accelerometer is comparable to waist-worn accelerometers. The wrist-worn Vivago® accelerometer is a waterproof device that does not require removal during the day. As a consequence subject compliance is improved and PA patterns in FLC are assessed more precisely. When a subject wears an accelerometer around the waist with an elastic belt or on a belt clip, the subject is obliged to remove the device for sleeping, changing clothes, doing contact sports, or during activities in water, e.g. bathing, showering, and swimming. These constraints may lead a lower compliance. Furthermore, when using waist-worn accelerometers, the zero activity periods of 20 min or longer are analysed as “not worn time” [20,21]. If these periods are removed from the total of activity it may lead to a misclassification of PA patterns, i.e. underestimation of sedentary time.

Controlled and noncontrolled activities were used in the study. While the majority of activities were of a controlled nature on a treadmill, two activities were not controlled, resting on a bed, and reading a book, where some participants were more active than others in spontaneous movements. The treadmill is a valid and reliable instrument for controlling different levels PA that is widely used in calibration studies [12-14]. A study showed that accelerometer output obtained on a treadmill was similar to data obtained on-land [22]. Consequently, the use of the treadmill in the present study can be extrapolated to estimate the portions of time at different levels of PA in adults under FLC.

A high correlation was found between data of the Vivago® accelerometer and oxygen consumption. In previous calibration and validation studies with others accelerometers (Actigraph®, RT3® accelerometer), significant correlations were observed between accelerometry and oxygen uptake. Therefore, the device was validated for assessing PA [13,17,23]**.** The correlation between accelerometer data and markers of PA intensity in the present study suggest that the Vivago® accelerometer is a valid instrument for measuring PA in adults. Moreover, this result is reinforced by the Bland & Altman method showing a good agreement in the assessment of energy expenditure (MET value) between the indirect calorimetry and the data obtained by the Vivago® wrist-worn accelerometer.

The Vivago® accelerometer is an original and innovative device in the assessment of PA in healthy or unhealthy populations. Its use is simple, fast and easy for data transfer. Moreover, the software interface is fun and allows for instant data interpretation. The device has possible application in treating a variety of important health concerns, as well as obesity.

This study provides useful information on the ability to the Vivago® wrist-worn accelerometer for assessing and detecting different activity levels. However, the study has some limitations. Firstly, the population was very homogeneous in age and physical fitness which may be a threat to external validity. Furthermore, the number of subjects was small and therefore less representative. To confirm our data, a second study is suggested with an independent group to validate the thresholds established in laboratory and field situations. Because of the limited number of devices used in the present study, we were unable to assess intra and inter-instrument reliability. The quality of accelerometers depends on their intra- and inter-instrument reliability. This is especially important for accelerometers because most studies of PA require the use of many different devices to simultaneously assess a large number of subjects. Therefore, intra and inter-instrument reliability is an important methodological issue for the choice of accelerometer device in a research study. Then, the lack of technical precisions on the Vivago accelerometer (data unavailable), specially for the equivalence in gravity force, can not permit to compare this accelerometer with the others (ActiGraph®, RT3®, Actical®) used widely in the literature.

## Conclusions

In conclusion, the Vivago® Wrist-Worn accelerometer is a valid technique used to assess PA in laboratory and FLC. This study provides cut-off points for adults for various levels of PA intensity using the Vivago® wrist-worn accelerometer measuring every minute. These thresholds enable quantification of the time spent performing sedentary, light, moderate, and vigorous activities for adults’ subjects. Further research is needed to assess inter and intra instrument reliability, a key indicator of the quality of the device. Further research should also determine if the thresholds established in laboratory can be extrapolated to field or FLC where activities are more unstructured, sporadic and with many interruptions during a typical day.

## Competing interests

The authors declare that they have no competing interests.

## Authors’ contributions

JV and RH performed the exams in this study and were involved in collecting, analyzing and interpretation the data and drafting the article. JM, GBX, and DT were involved in interpretation of the data and recruitment of subjects. PF were involved in revising article by providing significant advice and consultation. LB participated in the conception and design of the study, revising and editing the manuscript. All authors read and approved the final manuscript.

## Pre-publication history

The pre-publication history for this paper can be accessed here:

http://www.biomedcentral.com/1471-2458/12/690/prepub
